# Genetic and Molecular Factors Determining Grain Weight in Rice

**DOI:** 10.3389/fpls.2021.605799

**Published:** 2021-07-12

**Authors:** Ke Chen, Andrzej Łyskowski, Łukasz Jaremko, Mariusz Jaremko

**Affiliations:** ^1^Biological and Environmental Science and Engineering (BESE), King Abdullah University of Science and Technology (KAUST), Thuwal, Saudi Arabia; ^2^Rice Research Institute, Guangdong Academy of Agricultural Sciences, Guangzhou, China; ^3^Guangdong Key Laboratory of New Technology in Rice Breeding, Guangzhou, China; ^4^Faculty of Chemistry, Rzeszow University of Technology, Rzeszow, Poland

**Keywords:** rice, grain weight, grain size, regulatory mechanisms, molecular biotechnology

## Abstract

Grain weight is one of the major factors determining single plant yield production of rice and other cereal crops. Research has begun to reveal the regulatory mechanisms underlying grain weight as well as grain size, highlighting the importance of this research for plant molecular biology. The developmental trait of grain weight is affected by multiple molecular and genetic aspects that lead to dynamic changes in cell division, expansion and differentiation. Additionally, several important biological pathways contribute to grain weight, such as ubiquitination, phytohormones, G-proteins, photosynthesis, epigenetic modifications and microRNAs. Our review integrates early and more recent findings, and provides future perspectives for how a more complete understanding of grain weight can optimize strategies for improving yield production. It is surprising that the acquired wealth of knowledge has not revealed more insights into the underlying molecular mechanisms. To accelerating molecular breeding of rice and other cereals is becoming an emergent and critical task for agronomists. Lastly, we highlighted the importance of leveraging gene editing technologies as well as structural studies for future rice breeding applications.

## Introduction

Rice is one of the most important food crops globally. It is produced and consumed on a large scale, feeding nearly half the world population (Ashikari and Matsuoka, 2006; Kuroha and Ashikari, 2020). Therefore, research exploring the molecular mechanisms underlying rice yield production and stable high productivity is of great importance. Half a century of research initiated since the “green revolution,” which lasted from the 1950s to late 1960s, including the application of hybrid rice in the 1970s, has dramatically increased cereal production (Peng et al., 1999; Sasaki et al., 2002; Qian et al., 2016; Li S. et al., 2018). This period combined a set of technology initiatives focusing on both rice and wheat semi-dominant gene applications and cytoplasmic genetic male-sterile (CMS) line hybrid combinations. With continued population increases and the economic booms of the 21st century, the global population is estimated to reach over 9 billion by 2050 (Stamm et al., 2011). Food shortage caused by the contradiction between the boosting population and reduced arable land for farming, will become be a major challenge in every country. There are three main factors that determine single rice plant production: (i) effective panicles per plant, (ii) grain number per panicle, and (iii) grain weight. Years of research have culminated in the full rice genome sequence and annotation (Eckardt, 2000), including multiple functional genes controlled by major quantitative trait loci (QTLs), which were cloned and confirmed to affect rice phenotypes. Rice has been adopted by humans for more than 10,000 years and numerous variations exist among different rice species and sub-species. Differences in large-scale growth conditions have also led to great breeding difficulties (Huang et al., 2012). Recently, scientists have used map-based cloning, functional genomic analyses and Clustered Regularly Interspaced Short Palindromic Repeats (CRISPR) and CRISPR-associated endonuclease 9 (Cas9) gene editing to reveal many genes important for agronomic traits (Wang and Li, 2008; Zuo and Li, 2014; Hu et al., 2016). In this review, we summarize recent progress toward regulating grain weight, including QTLs and important monogenic loci for domestication and breeding. We conclude with the implications of these discoveries and discuss their applications using advanced molecular biological methods within the context of current global situations and trends. We also summarize and discuss how some proteins that have been characterized by structural biology approaches could accelerate molecular breeding in rice.

## Genetic and Molecular Determinants of Grain Weight Through Size Determination

Grain weight (thousand grain weight) is determined by grain length, grain width and grain thickness (Li et al., 2021). These traits affected grain weight so profoundly that they all shared potential trade-offs and coordinately control grain quantity and quality. Historically, grain size traits, including grain length, grain width and grain thickness, have been mainly investigated since the draft of rice genome map was released in Sasaki and Burr (2000). Nowadays, almost all the major QTLs relating to these traits have been reported, and several recent reviews have summarized these important traits (Zuo and Li, 2014; Fan and Li, 2019; Li N. et al., 2019; Li et al., 2021). Additionally, the development of glume strongly affects grain length and width. The filling process and endosperm development also affect the accumulation, conversion and starch synthesis of photosynthetic products. Over the last 20 years, cloning and functional studies of QTLs and mutant strains affecting grain size and endosperm development have provided the basis for understanding how these traits are regulated in rice.

Previous research has shown specific processes involved in regulating grain weight, such as ubiquitination, plant phytohormone regulation path-ways, G-protein regulation pathways, photosynthetic product accumulation, transportation and endosperm development processes, chromatin modifications, RNA-mediated regulation networks and others (Fan and Li, 2019; Li N. et al., 2019; Li et al., 2021). The term “grains” in rice has multiple meanings, including paddy rice (filled grains), brown rice or milled rice (Siebenmorgen et al., 2013). Here, we referred to “grains” as “filled grains” or “filled spikelets.” In our review, we focus on and summarize recent progress on the molecular regulatory mechanisms of coordination among the facets above in rice.

### Ubiquitination-Related Processes Involved in Grain Size Determination

Ubiquitin-mediated proteasome degradation is the main molecular process for digesting cellular proteins and is involved in most cellular metabolic processes *in vivo*. Ubiquitination involves three steps: activation, conjugation, and ligation. The ubiquitin-activating enzymes (E1s), ubiquitin-conjugating enzymes (E2s), and ubiquitin ligases (E3s) are involved in these steps, respectively (Komander and Rape, 2012). *Grain Width 2* (*GW2*) is a major QTL regulating grain width, and encodes a nuclear RING-type E3 ubiquitin ligase. Mutated *GW2* alleles cause an increase in cell size. Lines overexpressing *GW2* have reduced grain size. Combining Near Isogenic Lines (NIL) and overexpression line phenotypes suggest that GW2 is a negative regulator of cell enlargement (Song et al., 2007). GW2 interacts with chitinase14 (CHT14) and phosphoglycerate kinase (PGK), both of which are involved in carbohydrate metabolism by modulating their activity or stability (Lee et al., 2018). Additionally, the ubiquitin-specific protease15 (OsUBP15) physically binds to OsDA1, and genetic evidence shows that OsUBP15 also functions partially dependent with GW2 to regulate downstream substrates by ubiquitination and deubiquitination (Shi et al., 2019).

Deubiquitinases also regulate grain size. *WIDE AND THICK GRAIN 1* (*WTG1*) (also known as *OsOTUB1* and *GWC1*) encodes an otubain deubiquitinase with the same homolog domain of OTUB1 in humans (Saldana et al., 2019). Mutation of WTG1/GWC1 reduces its deubiquitin enzyme activity, leading to increased glume cell expansion and grain quality, and causing increased grain weight. In addition, OsOTUB1 interacts with IPA1/OsSPL14 via the proteasome regulation pathway. Loss of WTG1 function leads to accumulation of OsSPL14, which reduces tillering and increase yield production (Huang et al., 2017; Wang et al., 2017; Guo et al., 2020a).

Polyubiquitin binding protein can bind polyubiquitin and functions in the transfer process of substrate-polyubiquitin to the 26S proteasome. *Heading and grain weight* (*HGW*) encodes a novel plant-specific ubiquitin-associated (UBA) domain-containing nuclear and cytoplasmic protein that has polyubiquitin binding activity (such as ubiquitin-conjugating enzymes E2) and is co-expressed with multiple heading date and grain development-related proteins (Li et al., 2012). Mutations in ubiquitin ligase E3 and UBA proteins severely affect the development of testa and integument in Arabidopsis, suggesting the ubiquitin-mediated proteasome degradation pathway is well conserved across plant species (Li et al., 2008; Li and Li, 2014). We have summarized some of the genes related to the ubiquitin-mediated proteasome degradation process (Table 1).

**TABLE 1:** Lists of genes involved in ubiquitination and deubiquitination associated with grain size and grain weight.

**Gene**	**MSU name**	**Effects**			**Protein product**	**Citation**
		**Grain width**	**Grain length**	**Grain weight**		
*GW2*	LOC_Os02g14720	−−	−−	−−	E3 ubiquitin ligase	Song et al., 2007
*CHT14*	LOC_Os10g39680	N.A	N.A	N.A	Chitinase family protein	Lee et al., 2018
*PGK*	LOC_Os02g07260*	N.A	N.A	N.A	Phosphoglycerate kinase	Lee et al., 2018
*LG1*	LOC_Os02g14730	++	++	++	Ubiquitin specific protease 15	Shi et al., 2019
*WTG1/OTUB1/GMC1*	LOC_Os08g42540	−−	++	−−	Deubiquitinating enzyme	Huang et al., 2017; Wang et al., 2017; Guo et al., 2020a
*HGW*	LOC_Os06g06530	−−	NS	−−	Ubiquitin-associated domain protein	Li et al., 2012

“++”: significant positive correlation. “−−“: significant negative correlation. NS indicates no significance; N.A means not applicable. Star (*) indicates the potential MSU LOC number.

### Multiple Phytohormones Control Grain Weight

Phytohormones are involved in multiple plant activities such as growth, development, senescence, environmental adaptation, and stress responses. Almost all known phytohormones (auxin, ethylene, cytokinin, gibberellin, Brassinosteroids, and salicylic acid) regulate grain weight. These phytohormones function synergistically or antagonistically to regulate plant developmental processes. As expected, single mutations in phytohormone regulation pathway genes can alter multiple phenotypes.

#### Auxin

As the first identified plant hormone, Auxin is a plant growth hormone that promotes cell division and cell proliferation (Zhao, 2010). Studies have shown that auxin functions in multiple stages of plant growth, including seedling growth, gamete formation and embryo development (Wang et al., 2018). Polar Auxin Transport (PAT) and auxin signal transduction also affect grain development and maturation (Zhao, 2010). *Tillering and Small Grain 1* (*TSG1*) encodes a tryptophan amino-transferase that is an identical allele to the *FISH BONE* (*FIB*) gene (Yoshikawa et al., 2014; Guo et al., 2020b). *tsg1* mutants are hypersensitive to indole-3-acetic acid (IAA) treatment, and *TSG1* is dominant among four homologous genes in rice local auxin biosynthesis. VILLIN 2 (VLN2), an actin binding protein (ABPs), exhibits conserved actin filament bundling *in vitro*. *vln2* mutants have hypersensitive gravitropic responses as well as more rapid recycling of OsPIN2, eventually causing multiple phenotypes in architecture and wrinkled seeds (Wu et al., 2015). Another positive regulator of PAT is *Big Grain 1* (*BG1*), a universally expressed gene, with higher expression in shoots, spikelets and glumes. Increased expression of *BG1* enhances signal transduction of auxin and PAT, and eventually enlarges glumes and grain weight (Liu L. et al., 2015). *qTGW3*/*GL3.3*/*TGW3* is a negative regulator of grain size and a significant grain weight QTL that was discovered independently by three research groups. *qTGW3*/*GL3.3*/*TGW3* encodes a Glycogen Synthase Kinase 3/SHAGGY-like family protein kinase OsGSK5. It physically interacts with and phosphorylates Auxin Response Factor 4 (OsARF4). Moreover, GL3.3 interacts genetically with GS3, and in the genotypic combinations of *gs3gl3.3* in the recombinant inbred lines (RIL) they display significantly increased grain size (Hu Z. et al., 2018; Xia et al., 2018; Ying et al., 2018). *SMALL ORGAN SIZE 1* (*SMOS1*) and *SMALL ORGAN SIZE 2* (*SMOS2*) encode an auxin-regulated APETALA2-type transcription factor and a GA INSENSITIVE, REPRESSOR OF GAI, and SCARECROW (GRAS) transcription factor *DWARF AND LOW-TILLERING* (*DLT*), respectively. SMOS1 and SMOS2/DLT interact physically and form a keystone complex of Auxin-Brassinosteroids (BR) signaling crosstalk in rice (Aya et al., 2014; Hirano et al., 2017). Separately, the RING-finger and WD40-associated ubiquitin-like (RAWUL) domain-containing protein, Gnp4/LAX2, interacts with OsIAA3, and interferes with the OsIAA3-OsARF25 interaction to regulate grain length via the auxin signaling pathway. Additionally, OsARF25 binds the OsERF142/SMOS1 promoter and positively regulates OsERF142/SMOS1 gene expression (Zhang Z. et al., 2018). Another QTL, *GSA1*, encodes a UDP-glucosyltransferase that regulates grain size through flavonoid-mediated auxin levels and related gene expression. GSA1 is also required for the redirection of metabolic flux from lignin biosynthesis to flavonoid biosynthesis, which contributes to the regulation of grain size and abiotic stress tolerance (Dong et al., 2020).

#### Ethylene

Ethylene is the only small gas hormone produced in plants, which promotes leaf senescence, fruit maturation, seed dormancy and seedling triple reaction (Yin et al., 2017). EIN2 is a positive regulator of ethylene signaling in Arabidopsis. Lines overexpressing the EIN2 homolog gene *Mao Hu Zi 7* (*MHZ7*)/*OsEIN2* display enhanced coleoptile elongation, increased mesocotyl growth and larger grains in rice (Ma et al., 2013). *MHZ3* encodes an *endoplasmic reticulum* (ER) membrane protein. The MHZ3 protein is induced with ethylene treatment, and both the N- and C-terminus of the protein interact with OsEIN2, an N-terminal Nramap-like domain protein, inhibiting the ubiquitination of OsEIN2. MHZ3 overexpression significantly increases the protein density of OsEIN2 and increases rice grain weight. In contrast, *mhz3* mutants display insensitivity to ethylene and have smaller grains (Ma et al., 2018). Another key regulator in the ethylene signaling pathway is *OsEIL1*/*MHZ6*, the rice *EIN3* homolog. *mhz6* mutants exhibit insensitivity to ethylene and have reduced thousand grain weight (Yang et al., 2015). *Ethylene Receptor 2* (*ETR2*) encodes a serine/threonine kinase whose overexpression results in reduced grain weight (Wuriyanghan et al., 2009). These findings strongly suggest that ethylene sensitivity is positively correlated with grain weight and grain size. OsFBK12 encodes a Kelch repeat motif F-box protein that interacts with *Oryza sativa* S-phase Kinase-associated protein-like protein (OSK1). OsFBK12 promotes S-Adenosyl-L-methionine synthetase 1 (SAMS1) degradation by the 26S proteasome, leading to changes in ethylene levels and the regulation of leaf senescence and grain size (Chen Y. et al., 2013). Rice *spermidine/spermine synthase 1* (*OsSPMS1*) is a negative regulator in seed germination, grain size and grain yield. 1-aminocyclopropane-1-carboxylic acid (ACC) and ethylene content in seeds is significantly increased in RNA interfere (RNAi) lines and decreased in overexpression (OE) lines, respectively, suggesting *OsSPMS1* affects ethylene synthesis to regulate grain phenotypes in rice (Tao et al., 2018). These data indicate that genes contributing to ethylene regulation are involved to rice grain weight.

#### Cytokinin

Cytokinin (CK) promotes cell division and differentiation as well as the development of plant roots and shoots, apical dominance, senescence, fruit maturation, biotic and abiotic stress. The homeostasis of CK includes synthesis, activation, de-activation, re-activation and degradation. The key CK synthesis enzyme is isopentenyltransferase (IPT). Additionally, cytokinin oxidase/dehydrogenase (CKX) regulates CK degradation. CK expression *in vitro* increases grain size, an effect that is also achieved by reducing CKX expression (Jameson and Song, 2016). A drought and salt tolerant mutant, *dst*, was found to have a mutation in a zinc-finger transcription factor. DST binds to H_2_O_2_ metabolic-related gene promoters to regulate their expression and regulate drought and salt stress responses (Huang X. Y. et al., 2009). Moreover, DST directly binds to the promoter of *OsCKX2*. The semi-dominant allele *DST^reg1^* reduces expression of OsCKX2 in shoot apical meristems, resulting in the accumulation of CK in spikelet meristems. This results in enhanced cell division capacity and tremendously increases grain weight and number (Li et al., 2013). Guo et al. (2020c) verified that OsMAPK6 directly interacts with and phosphorylates DST to enhance the activation of OsCKX2. Overexpression of *IPT* under drought conditions delays drought stress responses and increases production yield (Peleg et al., 2011). *STRESS tolerance and GRAIN LENGTH* (*OsSGL*) encodes a Domain of Unknown Function (DUF) 1645 domain protein that positively responds to drought and salt stress. Overexpression of *OsSGL* alters expression of abiotic stress related and CK signaling marker genes, in addition to altering prolonged spikelet length, grain number per spike, grain length and grain weight phenotypes (Cui et al., 2016; Wang et al., 2016). A purine permease family gene, *OsPUP7*, is a positive regulator of CK transport and alters grain size and grain weight. In addition to OsPUP7, *Big Grain 3* (*BG3*) encodes *OsPUP4*, which is predominantly expressed in vascular tissues and is specifically suppressed by exogenous CKs. Differences in the localization of OsPUP7 and OsPUP4 suggest that these two proteins function in a linear pathway to direct cytokinin cell-to-cell transport and contribute to long-distance CK transport and local allocation. It also suggests that OsPIL15 regulates grain size by directly targeting OsPUP7 (Qi and Xiong, 2013; Ji et al., 2019; Xiao et al., 2019). CK distribution patterns are altered under salt stress, moving from the shoot to the root. Overexpression of *ARGONAUTE 2* (*AGO2*) activates *BG3* at an epigenetic level and mimics CK distribution patterns under salt conditions. This provides novel insight into the molecular balance between high yield production and abiotic resistance (Yin et al., 2020). Taken together, these results suggest that CK participates in critical abiotic stress tolerance responses and in young panicle development.

#### Gibberellins

Gibberellins (GAs) are a family of several phytohormones that are similar in structure. GAs promote seed germination, shoot elongation, leaf formation, flower power maturation, flowering induction and plant height (Kwon and Paek, 2016). *Grain Number per Panicle 1* (*GNP1*) encodes a GA biosynthesis gene *GA20 oxidase 1* (*GA20ox1*) and is a QTL referring to grain number and yield production in the rice variety TeQing (Wu et al., 2016b). GNP1 is negatively regulated by KNOX proteins, which modulate CK and GA activity in the meristem. It is worth noting that the *sd1* mutant is due to OsGA20ox2 mutations, a gene that results in the semi-dwarf architecture of the “green revolution” (Sasaki et al., 2002). *Small grain and dwarf 2* (*sgd2*) mutants show reduced plant height and grain size. *SGD2* was further confirmed by map-based cloning and complementation assays to be a homeodomain leucine zipper (HD-Zip) II family transcription factor. *SGD2* expression is predominantly detected in young panicles. *SGD2* regulates expression of many GA biosynthesis-related genes, suggesting HD-Zip transcription factors are involved in GA and other plant developmental processes (Chen et al., 2019c). Gibberellic acid stimulated transcript (GAST) family genes encode small polypeptides with a conserved cysteine-rich domain. In many plant species, GAST gene expression is induced following exposure to GA (Kumar et al., 2019). In rice, two GAST family member genes, *OsGASR9* and *GW6*, regulate grain size and yield and display positive responses to GA treatment. These findings suggest that GAST family genes have a conserved function for regulating the GA pathway and for contributing to grain and rice panicle developmental processes (Li X. et al., 2019; Shi et al., 2020). The GA response genes *Narrow Leaf 2* (*NAL2*) and *Narrow Leaf 3* (*NAL3*) encode WUSCHEL-related homeobox 3A (WOX3A) transcription factors, which negatively regulate GA biosynthesis and the homeostasis of GA and its precursors. Double mutant *nal2nal3* displays multiple developmental abnormalities, including increased tillers, decreased later roots and smaller grain size. Plant height is also negatively affected. Overexpression of *OsWOX3A* mirrors GA defective phenotypes, including short plant height and thinner leaves as well as lower grain number and smaller grain size (Cho et al., 2013, 2016). Synthesis of GA regulation pathway genes is negatively regulated by Calcium-Dependent Protein Kinase 1 (OsCDPK1). *OsCDPK1-RNAi* plants show increased plant height, grain width and thousand grain weight, while overexpression lines show the opposite phenotypes (Ho et al., 2013). *Short Grain Length* (*SGL*) encodes a kinesin-like protein with transcriptional activation activity. Mutation of *SGL* alters GA metabolism related gene expression, leading to dwarf size, shorter spikelet and shorter glume cell length phenotypes (Wu et al., 2014).

#### Brassinosteroid (BR)

Isolated from 1979, BR was identified from pollens of *Brassica napus* with steroids analogous to the steroid hormones present in animals (Salvi et al., 2021). BR functions in seed germination, stomatal formation, vascular bundle differentiation, flowering fertility and senescence, all of which affect important agronomic traits like plant height, leaf angles, grain size and tiller numbers (Zhang et al., 2014). Normally, mutation of a related gene in the BR regulation pathway always alters grain size traits, causing smaller and circle shape grains. Within the BR biosynthesis pathway, mutation of cytochrome P450 key enzyme *DWARF 11* (*D11*) (Tanabe et al., 2005; Wu et al., 2016a; Zhou et al., 2017), BR C-6 oxidase (*OsBR6ox*)/*BR*-*deficient dwarf 1* (*BRD1*) (Hong et al., 2002), *DWARF 2* (*D2*) (Hong et al., 2003; Fang et al., 2016) and other BR biosynthesis-related genes such as *BRD2* (Hong et al., 2005) all cause similar phenotypes, including reduced plant height, shoot length, spikelet length and grain size. Gain-of function mutations in *slender grain Dominant* (*slg-D*) result in a less compact plant architecture, with larger leaf angles and significantly longer grain length. *SLG*-*RNAi* plants display BR defective phenotypes, including shorter and more circular grain shape, smaller leaf angles and dwarf plants. Overexpression of *SLG* in *d11* and BR signaling reduction in the mutant *d61* do not restore BR defective phenotypes, suggesting SLG regulates rice grain and leaf angles through the BR biosynthesis pathway (Feng et al., 2016). Mutations in BR signaling transduction-related genes also results in phenotypes similar to those above. *GW5* encodes a calmodulin binding protein and is a major QTL for grain width (Duan P. et al., 2017). In the Nipponbare rice variety, a 1,212 bp deletion located 5.7 kb upstream of GW5 results in lower *GW5* expression and increases grain width. Grain width is also dramatically reduced in *GW5* overexpression lines. Moreover, GW5 directly interacts with a key kinase, Glycogen Synthase Kinase 2 (GSK2), during BR signaling and inhibits its enzymatic activity. GSK2 downstream transcription factors BZR1 and DLT then enter the nucleus in an unphosphorylated state to modulate BR signaling response genes and ultimately affect rice grain size (Shomura et al., 2008; Weng et al., 2008; Liu et al., 2017). Additional evidence suggests GW5 and GW5-like proteins physically interact with BR signaling key components GSK2 and BIN2, resulting in accumulation of unphosphorylated DLT and OsBZR1 (Liu et al., 2017; Tian et al., 2019). GSK2 also interacts with and phosphorylates OVATE family protein 8 (OFP8) (Yang et al., 2016). It is also notable that OFP8 and the OVATE family protein OFP14 interact with each other as well as the transcriptional activator GS9. Transcriptional activity of GS9 is reduced by OFP14 and overexpression of GS9 results in BR-defective phenotypes such as circular grain shape. This suggests that GS9 is a negative regulator of BR signaling (Zhao et al., 2018). In addition to OFP8 and OFP14, OFP3 interacts with both GSK2 and DLT in yeast two-hybrid screens and acts as a negative regulator of the BR response (Xiao et al., 2020). OFP1 interacts with DLT and GSK2 and its promoter is regulated by OsBZR1, which functions as an additional positive regulator of BR signaling (Xiao et al., 2017). Beyond OFP family proteins, MEI2-LIKE PROTEIN 4 (OML4) is phosphorylated downstream by GSK2, suggesting that the GSK2-OML4 regulatory module of grain size is a suitable target for breeding (Lyu et al., 2020).

**FIGURE 1:**
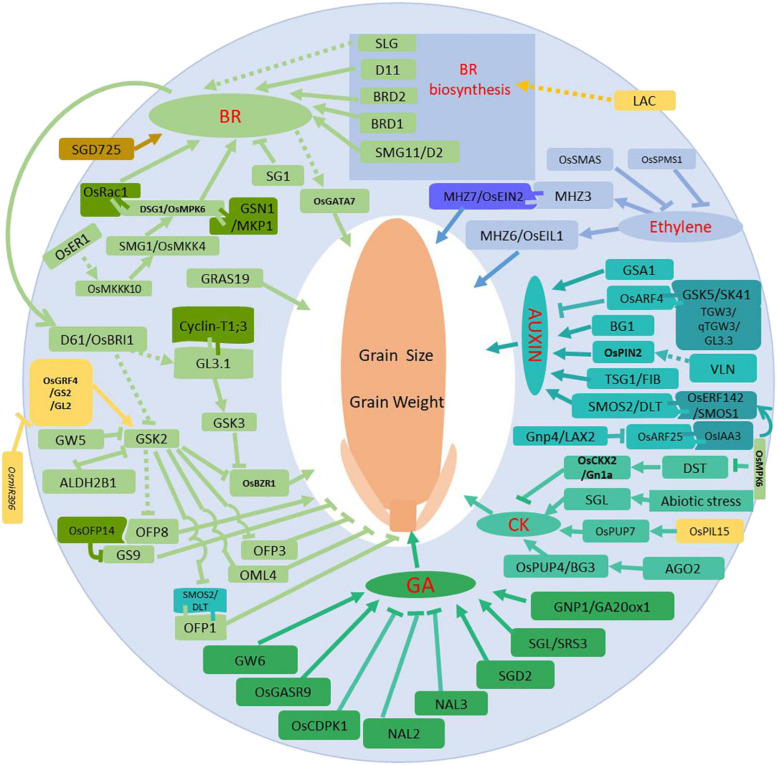
Regulation network via phytohormone processes for grain size and grain weight. Regular arrows indicate activated or positive regulation, and block arrows indicate inhibition or negative regulation. The solid lines are supported by solid scientific evidence, and the dotted lines represent hypothetical associations. Red lettering refers to the phytohormones, and different colors show proteins that are involved in different phytohormone transduction signaling pathways.

In addition to the described BR signaling members, novel and still unknown mechanisms are involved in BR signal transduction. *D1/RGA1* encodes an α subunit of a heterotrimer G-protein and *d1* mutants are insensitive to BR treatment, displaying short shoots and small grains (Wang et al., 2006). *Taihu Dwarf 1* (*TUD1*) encodes a U-box containing E3 ubiquitin ligase that directly interacts with D1/RGA1 and mediates BR signal transduction to modulate grain size. *d1tud1-5* double mutants show shorter internodes, reduced leaf angles and smaller grain sizes, similar to *tud1-5* mutants. This suggests that *TUD1* functions upstream of *D1* (Hu et al., 2013). GATA factors are a large family of transcriptional regulators in eukaryotes. GATA factors feature zinc finger motifs and DNA binding domains. *OsGATA7* artificial microRNA and CRISPR/Cas9 knockout plants are insensitive to BR treatment, while overexpression lines are similar to wild-type plants. BR induces expression of OsGATA7, suggesting OsGATA7 may be involved in the regulation of rice grain size. However, the regulation of OsGATA7 cellular levels more closely resembles classical BR regulation, suggesting OsGATA7 is also involved in other signaling pathways (Zhang Y. J. et al., 2018). SHORT GRAIN 1 (SG1) encodes a putative protein that is expressed in roots and young panicles. Gain-of-function *Sg1-D* mutants increase SG1 expression and reduce BR sensitivity. Interestingly, while BR content remains constant, cell divisions and cell numbers are reduced, leading to dwarf plants and smaller grain size phenotypes (Nakagawa et al., 2012). OsALDH2B1 functions within the biological trade-off between growth and defense. OsALDH2B1 functions primarily as an aldehyde dehydrogenase and secondarily as a transcriptional regulator. OsALDH2B1 antagonizes OsBZR1 function together with the Jasmine Acid (JA)-related immune gene *AOS2*, where both genes are direct targets of OsBZR1 (Ke et al., 2020). GRAS family genes are putative environmental signaling genes. Two studies confirmed OsGRAS19 involvement in BR signaling and suggested OsGRAS19 acts downstream of OsBES1 (Chen L. et al., 2013; Lin et al., 2019). BR signaling is strongly associated with the Mitogen Activated Protein Kinases (MAPK) pathway for regulating grain size. *SMALL GRAIN 1* (*SMG1*) encodes Mitogen Activated Protein Kinase Kinase 4 (OsMKK4) in rice. *smg1* mutant phenotypes include shorter plant height, smaller leaf angles, shorted spikelet and grain length, which are a similar set of phenotypes as BR mutants. *smg1* mutants are also insensitive to BR treatment and have reduced RNA expression levels of BR signaling-related genes, such as *D61*/*BRI1*, *BZR1*, *GSK2*, and *DLT* (Duan et al., 2014). *Dwarf and Small Grain 1* (*DSG1*) encodes a Mitogen Activated Protein Kinase 6 (MAPK6) with phosphorylation enzyme activity. OsMAPK6 strongly interacts with OsMKK4 and functions as a kinase downstream of OsMKK4 to modulate target gene expression. Similar to *smg1* mutants, *dsg1*/*mapk6* mutants are also insensitive to BR treatment, and display phenotypes similar to *smg1* mutants (Liu S. et al., 2015). MAPK cascade source protein Mitogen Activated Protein Kinase Kinase Kinase 10 (OsMKKK10) interacts with and phosphorylates OsMKK4 to control grain size. Loss-of-function *osmkkk10* mutants have smaller grains, lower grain weights and are dwarfs. In contrast, the OsMKKK10 constitutive overexpression line (CA-OsMKKK10) shows enlarged grain sizes and spikelets. Biochemical and genetic analyses suggest that OsMKKK10, OsMKK4, and OsMAPK6 work together to modulate grain size (Xu et al., 2018). *GRAIN SIZE AND NUMBER 1* (*GSN1*) encodes Mitogen Activated Protein Kinase Phosphatase (OsMKP1), which is a dual-specificity phosphatase of unknown function. GSN1 directly interacts with and dephosphorylates OsMAPK6. GSN1 also participates in the GSN1-MAPK module and functions as a negative regulator of grain size (Guo et al., 2018). Two research groups have confirmed these results in different rice varieties (Wang T. et al., 2019; Zhang X. et al., 2019). Moreover, a well-known Receptor-Like kinase (RLK), *OsER1*, was identified to act upstream of the OsMKKK10-OsMKK4-OsMAPK6 cascade to regulate spikelet number in rice. The article also reports the important interaction between OsMAPK6 and DST, genetically finalizing the regulation network among MAPKs and cytokinin (Guo et al., 2020c). In addition to the interactions of DST and OsMAPK6, interactions between OsRac1 and OsMAPK6 also regulate grain size. *OsRac1* encodes a ROP GTPase, whose homolog in Arabidopsis and rice contributes to plant growth and development as well as disease resistance. OsRac1 also acts as a positive regulator of rice grain size (Zhang Y. et al., 2019). *qGL3*/*GL3.1* is a major QTL that controls grain length grain, weight and grouting. *qGL3*/*GL3.1* encodes a Kelch repeat serine/threonine protein phosphatase OsPPKL1 (Hu et al., 2012; Qi et al., 2012; Zhang et al., 2012). A D364E amino acid change within the second conserved Kelch repeat AVLDT motif alters phosphatase activity. Wild type qGL3/GL3.1 protein has higher phosphatase activity toward the substrate Cyclin-T1;3, which alters grain length by making changes to the cell cycle. Recently it was shown that qGL3/GL3.1 interacts with the GSK3/SHAGGY-Like Kinase OsGSK3 to modulate BR signaling (Gao et al., 2019). Taken together, these results suggest that BR signaling, MAPK signaling and other signaling pathways use phosphorylation for specifically regulating grain size and to coordinate a trade-off of plant developmental priorities. We have generated a figure to show all the main genes and their roles in phytohormone processes in association with grain weight (Figure 1).

### G-Protein Processes Regulate the Central Hub MADS1/LGY3 to Affect Grain Traits

**FIGURE 2:**
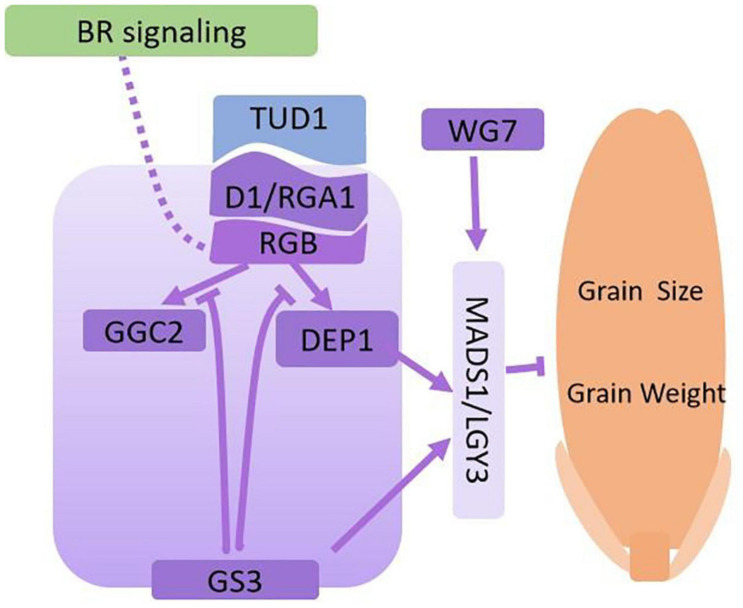
Regulatory network via G-protein processes for grain size and grain weight. The dotted line from BR signaling to G-proteins shows the potential regulatory roles. The regular arrows indicate the activation or positive regulation, and the block arrows indicate inhibition or negative regulation. The solid lines show interactions supported by solid scientific evidence, and the dotted lines reprensent hypothetical associations. The purple color boxes indicates the proteins that all involved in G-protein signaling.

Previously, Hu et al. (2013) described how the α subunit of G-protein (*D1*/*RGA1*) regulates grain size through the BR signaling pathway. This result emphasized that BR signaling might act upstream of the G protein regulation network. However, other subunits of G-proteins are also involved in regulating grain size. *RGB1* encodes heterotrimeric G protein β-subunit (Gβ). *RGB1-RNAi* plants are dwarfs and have smaller grain sizes (Utsunomiya et al., 2011). *Grain Size 3* (*GS3*) is a major QTL for grain size and encodes a γ subunit of the G-protein transmembrane protein that contains an Organ Size Regulation (OSR) domain. *gs3* mutants or *GS3*-*RNAi* knockdown lines have significantly enlarged grain size (Fan et al., 2006; Mao et al., 2010). *DEP1*, similar to *GS3*, encodes another OSR domain-containing protein and is a major QTL for grain number. However, DEP1 has the opposite function to GS3, as DEP1 overexpression lines have fewer grain numbers with larger grain size (Huang X. et al., 2009; Sun et al., 2018). LGY3 is a QTL that negatively regulates grain length. *LGY3* encodes a MADS family transcription factor OsMADS1. Deletion of the C-terminal transcription domain of OsMADS1 reduces its transcriptional activity, and increases grain length. OsMADS1 regulates grain size as a downstream member of G-protein signaling transduction through interaction with GS3 and DEP1 (Liu et al., 2018; Sun et al., 2018). *WIDE GRAIN 7* (*WG7*) encodes a cysteine-tryptophan (CW) domain-containing transcriptional activator that directly binds the CATTTC motif within the OsMADS1 promoter to alter H3K4me3 levels (Huang et al., 2020). The protein GGC2 shares 66 and 48% protein similarity to DEP1 and GS3, respectively, and is also a G-protein γ subunit involved in regulating rice grain size. Overexpression of GGC2 increases grain length, while GGC2 knockout plants have significantly reduced grain length, similar to the regulation pattern of DEP1. Additional evidence suggests GS3, as a G protein γ subunit, competes for the binding of G-protein α and β subunits (RGA1/GRB complex), decoupling DEP1 and GGC2 to block downstream signal transduction. Interestingly, the GS3 C-terminal domain mediates its own degradation to modulate G protein signal transduction (Sun et al., 2018). A simple working models showing how G protein processes are involved in grain weight regulation in rice is shown in Figure 2.

### Photosynthetic Product Accumulation, Transportation and Endosperm Development Processes Regulated Grain Size, Weight and Quality

The hull is the predominant factor determining rice grain weight and is a storage source for photosynthetic products in rice. Back in 1970, Takeda and Takahashi proposed that the dimension of the hull determines the grain size and shape (Takeda and Takahashi, 1970). This concept also implies that genes that regulate cell elongation and cell expansion in spikelet hulls functioning synchronously with endosperm development-related genes contribute to grain size, as discussed in certain reviews (Li N. et al., 2018; Azizi et al., 2019). The distribution, accumulation and transportation of photosynthetic products affect grouting and seed quality, while the development and maturation of endosperm contribute to grain weight. *QUASIMODO2* (*OsQUA2*) encodes a putative pectin methyltransferase. OsQUA2 is essential for maintaining a high degree of methylesterification of homogalacturonan in rice culm-sieve element cell walls and provides channel support for the long distance “source” to “sink” transport of photosynthetic products (Xu et al., 2017). Vacuolar invertases OsINV2 and OsINV3 positively regulate starch constitution and sugar metabolism in rice, and OsINV3 has been proven as a major regulator of grain size via sink organ sucrose metabolism (Deng et al., 2020). *OsSWEET4* encodes a sucrose transport protein that controls hexose import from the maternal phloem. *ossweet4* mutants have reduced hexose transport ability and diminished endosperm, resulting in hindered grouting and reduced grain weight. OsSWEET4 has a positive feedback on grouting through the induction of glucose (Sosso et al., 2015). The ethylene receptor ETR2 regulates photosynthetic product distribution by inhibiting α-amylase expression to alter grain grouting. Overexpression of ETR2 significantly reduces *RAmy3D* expression and transformed lines accumulate starches in their shoot and internode instead of their grains (Wuriyanghan et al., 2009). A membrane localized protein, GRAIN-FILLING RATE 1 (GFR1), interacts with the Rubisco small unit by yeast two-hybrid assay, suggesting it functions to regulate rice grain-filling rate by promoting sucrose uploading (Liu et al., 2019).

The starch granule is the main component in endosperm. Starch granules are formed by two polymers, amylose and amylopectin. The biosynthesis and ratio of amylose and amylopectin determines grain filling and quality. *GRAIN INCOMPLETE FILLING 1* (*GIF1*) encodes a cell-wall invertase that is required for carbon partitioning during early grain filling. *gif1* mutants display slowed grouting and grain chalkiness, while overexpression of *GIF1* increases grain length and weight (Wang et al., 2008). *Floury endosperm 2* (*FLO2*) encodes a novel tetratricopeptide repeat domain protein that is conserved in plants and regulates amylose concentration. *flo2* mutant grains have a significantly increased chalkiness percentage with reduced expression of starch biosynthesis and sucrose synthase genes. Overexpression of *FLO2* in rice significantly increases grain size and thousand grain weight (She et al., 2010). Another floury endosperm mutant, *flo7*, displays floury periphery phenotypes around the endosperm in addition to significantly reduced amylose concentrations. Alteration of amylopectin in *flo7 lines* also affects grain weight and quality. *FLO7* encodes a DUF1388 protein that is specifically expressed in the endosperm periphery and is required for starch synthesis and amyloplast development (Zhang et al., 2016). *Pyruvate Kinase 2* (*OsPK2*) encodes a plastidic pyruvate kinase involved in rice endosperm starch synthesis, compound granule formation and grain filling. Furthermore, OsPK2 (PKpα1) and three other putative rice plastidic isozymes, PKpα2, PKpβ1, and PKpβ2, interact to form a heteromeric complex for maintaining metabolic homeostasis in plastids (Cai et al., 2018). In addition to starch biosynthesis genes that affect grain quality, additional genes contribute to starch granule development. FLOURY ENDOSPERM 16 (FLO16) encodes an NAD-dependent cytosolic malate dehydrogenase. The *flo16* mutant shows a large reduction in ATP content leading to a reduction in starch synthesis-related enzyme activity (Teng et al., 2019). Previous research reported that Heat Shock Proteins (HSPs) also contribute to starch granule development. *FLO11-2* encodes a plastid-localized cpHSP70-2 that contributes to chalkiness in a temperature-dependent manner (Tabassum et al., 2020). *FLO10* encodes a pentatricopeptide repeat protein that acts as a chaperone in the *trans*-splicing of mitochondrial *nad1* intron 1 during endosperm development (Wu et al., 2019). It has also been shown that a mitochondrial complex I subunit (FLO13) controls grain size, grain quality and thousand grain weight and is associated with the respiratory electron chain complex of the mitochondria (Hu T. et al., 2018). A class I glutamine amidotransferase named FLO19, has been reported to affect grain quality and the expression of other amyloses (Lou et al., 2021).

Nutritive endosperm of seeds is a unique feature of angiosperms that is initiated by double fertilization. During the development of nucleotype, the nucleus of the primordial endosperm undergoes nuclear division to produce multinucleated cells. However, this is not accompanied by the production of cell walls during nuclear development. Subsequent cellularization occurs during the cell phase. Timing of the transformation from the syncytial stage to cellularity is critical for endosperm development and eventual seed formation (Brown and Lemmon, 2007). *THOUSAND-GRAIN WEIGHT 6* (*TGW6*) encodes an IAA-glucose hydrolase that regulates endosperm development and grain grouting processes by controlling IAA supply and increasing translocation rates from source organs in sucrose metabolism (Ishimaru et al., 2013). In the NIL, a loss of function frameshift *tgw6* allele leads to a significant reduction in the amount of free IAA. OsNF-YB1, a member of the nuclear factor Y (NF-Y) gene family, is specifically expressed in endosperm as a histone-like transcription factor and functions in endosperm cell proliferation. *OsNF-YB1-RNAi* transformed plants and *osnf-yb1* mutants both exhibit increased chalkiness rate in grains, as well as reduced grain width, thickness, and amylose concentration. OsNF-YB1 directly binds promoters containing GCC box ERF transcription factors (Sun et al., 2014; Bai et al., 2016; Xu et al., 2016). Furthermore, OsNF-YB1, OsNF-YC12, and bHLH144 form a complex to maintain NF-YB1 stability and protect NF-YB1 from degradation by the ubiquitin/26S proteasome. OsNF-YC12 regulates grain quality by directly binding the *FLO6* and *Glutamine Synthetase 1;3* (*OsGS1;3*) promoters (Peng et al., 2014; Bello et al., 2019; Xiong et al., 2019). Further evidence suggests nuclear factor Y functions in rice grain quality (E et al., 2018; Bello et al., 2019). OsNF-YC10, OsNF-YB9, OsNF-YC2, and OsNF-YC4 contribute to grain size and panicle development (Kim et al., 2016; Das et al., 2019; Jia et al., 2019). These results suggest nuclear Y factors tightly regulate flower organogenesis. Moreover, other proteins such as starch branching enzyme (OsBEIIB), isoamylase (ISA1), natural resistance associated macrophage proteins (NRAMP) family 5 (OsNramp5), and ADP-glucose transporter (OsBT1) etc., also showed positively associate with the development of rice quality (Yang et al., 2012; Li S. et al., 2017; Sun et al., 2017; Tang et al., 2017; Fiaz et al., 2019; Shufen et al., 2019). Table 2 below shows some of these genes involved in photosynthetic related processes (Table 2).

**TABLE 2:** List of genes that function via photosynthetic product accumulation, transportation, and endosperm development processes in grain weight and size.

**Gene**	**MSU name**	**Grain size**	**Grain quality**	**Thousand grain weight**	**Protein product**	**References**
*QUA2*	LOC_Os02g51860	N.A	N.A	++	Pectin methyltransferase	Xu et al., 2017
*OsINV3*	LOC_Os02g01590	++	N.A	++	Vacuolar invertase	Deng et al., 2020
*SWEET4*	LOC_Os02g19820	++	++	N.A	Glucose and fructose transporter	Sosso et al., 2015
*ETR2*	LOC_Os04g08740	N.A	−−	−	Ser/Thr kinase, ethylene receptor	Wuriyanghan et al., 2009
*GFR1*	LOC_Os10g36400	NS	+	++	Putative membrane protein	Liu et al., 2019
*GIF1*	LOC_Os04g33740	N.A	++	++	Cell wall invertase	Wang et al., 2008
*FLO2*	LOC_Os04g55230	+	++	++	Tetratricopeptide repeat (TPR) motif protein	She et al., 2010
*FLO7*	LOC_Os10g32680	NS	++	+	DUF1388 protein	Zhang et al., 2016
*PK2*	LOC_Os07g08340	++	++	N.A	Plastidic pyruvate kinase	Cai et al., 2018
*FLO16*	LOC_Os10g33800	+	++	++	Malate dehydrogenase	Teng et al., 2019
*FLO11-2*	LOC_Os12g14070	N.A	++	N.A	Plastid-localized cpHSP70-2	Tabassum et al., 2020
*FLO10*	LOC_Os03g07220	++	++	++	P-type PPR protein	Wu et al., 2019
*TGW6*	LOC_Os06g41850	−−	NS	−−	IAA-glucose hydrolase	Ishimaru et al., 2013
*FLO13*	LOC_Os02g57180	++	++	++	Mitochondrial complex I subunit	Hu T. et al., 2018
*FLO19*	LOC_Os03g48060	+	++	++	Class I glutamine amidotransferase	Lou et al., 2021
*NF-YC12*	LOC_Os10g11580	++	++	++	Nuclear transcription factor Y subunit C12	Bello et al., 2019; Xiong et al., 2019
*FLO6*	LOC_Os03g48170	N.A	++	N.A	CBM48 domain-containing protein	Peng et al., 2014
*NF-YC10*	LOC_Os01g24460	++	N.A	++	Nuclear transcription factor Y subunit C10	Jia et al., 2019
*NF-YB9*	LOC_Os06g17480	N.A	N.A	++	Nuclear transcription factor Y subunit B9	Das et al., 2019
*NF-YC2*	LOC_Os03g14669	N.A	N.A	N.A	Nuclear transcription factor Y subunit C2	Kim et al., 2016
*NF-YC4*	LOC_Os06g45640	N.A	N.A	N.A	Nuclear transcription factor Y subunit C4	Kim et al., 2016
*OsBEIIb*	LOC_Os02g32660	N.A	++	N.A	Starch branching enzyme	Yang et al., 2012; Sun et al., 2017
*ISA1*	LOC_Os08g40930	++	++	++	Isoamylase	Shufen et al., 2019
*OsNramp5*	LOC_Os07g15370	NS	N.A	NS	(NRAMP) family 5	Tang et al., 2017
*OsBT1*	LOC_Os02g10800	NS	++	++	ADP-glucose transporter	Li S. et al., 2017

*“++”: significant positive correlation. “−−”: significant negative correlation. NS indicates no significance; N.A means not applicable.*

### Chromatin Modification Processes Participate in Grain Characterization

Epigenetics is the study of heritable phenotype changes that do not arise from alterations in DNA sequence (Dupont et al., 2009). These chromatin modification processes include DNA methylation and acetylation or methylation of lysine residues on histones H3 and H4. In the past 20 years, many novel discoveries have shown how epigenetic processes shape plant development and abiotic stress tolerance.

*Grain Weight on chromosome 6a* (*GW6a*) was the first grain size QTL shown to act through epigenetic mechanisms. *GW6a* encodes a GNAT-like histone acetyltransferase (*Osg1HAT1*) with a conserved GNAT motif. This GNAT motif is a conserved acetyl-CoA (CoA) binding site for acetyltransferases. Overexpression of *Osg1HAT1* increases glume cell number, grain grouting rate, grain size and thousand grain weight. Additionally, Osg1HAT1 overexpression also increases histone H4 acetylation, resulting in gene expression changes for phytohormone responses, protein metabolism, the cell cycle and other physiological processes (Song et al., 2015).

Lysine methylation of histones is also vital for forming heterochromatin and regulating gene expression. SET domains function as histone lysine methyltransferases. *SET Domain Group 728* (*SDG728*) is a homolog of H3K9 methyltransferase *Su* (*var*)*3-9* in *Drosophila*. Reduced SDG728 expression causes a global reduction of H3K9 methylation levels and leads to smaller grain size. Evidence suggests that SDG728 inhibits transposon activity, including transposon *Tos17* activity (Qin et al., 2010). SDG725 encodes a H3K36 methyltransferase. The *sdg725* mutant and *SDG725-RNAi* plants exhibit phenotypes mirroring those of BR defects, including dwarf size, smaller leaf angles and shorter grains. SDG725 directly binds to key genes involved in BR synthesis and signal transduction and mediates H3K36 histone methylation involved in modulating the BR pathway (Sui et al., 2012).

Semi-dominant *Epi-rav6 mutants* display enlarged leaf angles, dwarf size, and have smaller grain size. They also upregulate expression of BR biosynthesis and transduction-related genes such as *BRI1*, *BRD1*, *D11*, and *D2*. BR biosynthesis inhibitors can also restore large leaf angle phenotypes in *rav6* mutants. Bisulfite analysis revealed that cytosine methylation of four CGs and two CNGs within a continuous 96-bp region is critical for regulating RAV6 expression (Zhang et al., 2015).

Fertilization-Independent Endosperm 2 (OsFIE2) is a member of the PcG family of proteins. OsFIE2 possesses specific histone H3 methyltransferase activity and controls H3K27me3 formation. Downregulation of *OsFIE2* results in various phenotypes, including smaller grain size, lower fertility rate and pre-germination. Phytohormone biosynthesis and signaling transduction-related genes, as well as starch synthesis and cell division genes, are also affected by OsFIE2 expression (Nallamilli et al., 2013; Liu et al., 2016). Interestingly, tissues that lack *OsMADS6* expression contain H3K27me3 modifications, while tissues with OsMADS6 expression, including flower organs and endosperm, have H3K36me3 modifications. The *osmads6* mutant displayed multiple phenotypes, including larger grain width, smaller grain length, lower starch concentration, higher grain protein concentration and abnormal flower development. It remains unknown whether OsFIE2 mediates H3K27me3 formation in the OsMADS6 promoter (Zhang et al., 2010). Unlike OsFIE2, which is constitutively expressed the homolog protein OsFIE1 is only specifically expressed in endosperm. The mutant *osfie1* produced smaller seeds, and OsFIE1 overexpression lines had increased mature grain size under high night temperature (HNT). Although both OsFIE proteins exhibited H3K27me3 methyltransferase activity in plants, OsFIE2 has a more important function than OsFIE1 protein during rice endosperm development (Cheng et al., 2020; Dhatt et al., 2021). The table below lists the main genes participating in epigenetic processes regulating grain traits (Table 3).

**TABLE 3:** Lists of genes functioning in grain weight via chromatin modification processes in grain weight and size.

**Gene**	**MSU name**	**Grain width**	**Grain length**	**Thousand grain weight**	**Protein product**	**Epigenetic modification**	**References**
*GW6a*	LOC_Os06g44100	++	++	++	GNAT-like histone acetyltransferase	H4 acetylation	Song et al., 2015
*SDG728*	LOC_Os05g41172	N.A	N.A	+	SUVH Histone Methyltransferase	H3K9 methylation	Qin et al., 2010
*SDG725*	LOC_Os02g34850	N.A	N.A	++	H3K36 methyltransferase	H3K36 methylation	Qin et al., 2010
*RAV6*	LOC_Os02g45850	−−	−−	−−	B3 DNA binding domain	N.A	Zhang et al., 2015
*FIE2*	LOC_Os08g04270	++	++	++	Polycomb Protein	H3K27 methylation	Nallamilli et al., 2013; Liu et al., 2016
*MADS6*	LOC_Os02g45770	+	+	NS	MADS-box protein	N.A	Zhang et al., 2010
*FIE1*	LOC_Os08g04290	−−	NS	−−	Polycomb Protein	H3K27 methylation	Cheng et al., 2020; Dhatt et al., 2021

*“++”: significantly positive correlation. “+”: positive correlation. “−−”: significantly negative correlation. NS: no significance; N.A: not applicable.*

### MicroRNA Processes Regulate Grain Traits by Targeting Expression of Specific Genes

MicroRNAs are small non-coding RNAs that universally occur in eukaryotes. MicroRNAs demonstrate conserved post-transcriptional regulation functions through their ability to bind target mRNAs and mediate their translation initiation or degradation. MicroRNAs participate in many plant activities including organism development, biotic and abiotic responses, environmental adaptation and fruit senescence (Chen, 2009; Tang and Chu, 2017). In this section, we summarize the contribution of microRNAs to grain weight.

#### *OsmiR156* and Its Regulation Network

The first microRNA found to regulate grain weight and yield was *OsmiR156*. *Ideal Plant Architecture 1* (*IPA1*) is a QTL that encodes an SPL family transcription factor, OsSPL14, which controls rice shoots and grains. *OsSPL14-RNAi* transformed plants display dwarf size, narrow shooting, fewer branches and reduced production. *OsSPL14* is the gene targeted by miR156 and is mostly expressed in shoot tips, primary branches and secondary branches, in contrast to OsmiR156 expression. Overexpression of OsmiR156 significantly reduces OsSPL14 mRNA levels, while antisense RNA MIM156 increases expression of OsSPL14. This suggests that *OsmiR156* directly regulates OsSPL14 expression levels by mediating the degradation of *OsSPL14* transcripts. In the *japonica* line Shaoniejing (SNJ) rice variety, a point mutation in exon three of OsSPL14 abolishes OsmiR156 binding and mediates the degradation of OsSPL14. This results in reduced tiller number, enhanced lodging resistance, larger spikelets and increased thousand grain weight (Jiao et al., 2010; Miura et al., 2010). Rice *SHORT INTERNODES 1* (*OsSHI1*) encodes a plant-specific transcription factor of the SHI family and contains a characteristic family specific IGGH domain. OsSHI1 also contains a conserved zinc-finger DNA binding domain that physically interacts and acts antagonistically with IPA1 protein *in vivo* and *in vitro*. This interaction expands the OsSPL protein family regulation network to control plant architecture and grain size (Duan et al., 2019). *Grain Width 8* (*GW8*) is a major QTL that encodes the OsSPL16 transcription factor. OsSPL16 positively regulates the G1 phase to S phase cell cycle transition and regulates cell division-related genes. OsSPL16 also accelerates cell division and the grain grouting process and increases grain width and yield production. Similar to OsSPL14, OsSPL16 is another target gene of OsmiR156. Overexpression of OsmiR156 significantly reduces OsSPL16 expression resulting in reduced rice grain size and thousand grain weight (Wang et al., 2012). *Grain Length on Chromosome 7* (*GL7*)/*Grain Width 7* (*GW7*) is a QTL controlling grain size and grain weight. GL7/GW7 promotes cell length in glume and reduces chalkiness percentage in endosperm, both of which improve grain quality. OsSPL16/GW8 is a transcription factor that directly binds the promoter of *GL7*/*GW7* and inhibits its expression (Wang S. et al., 2015; Wang Y. et al., 2015). *GRAIN LENGTH AND WEIGHT ON CHROMOSOME 7* (*GLW7*) was the first grain size QTL identified by genome-wide association (GWAS) studies. GLW7 encodes the SPL family member OsSPL13. GLW7 significantly increases glume cell length, which subsequently increases grain weight and yield production. The 3-prime-untranslated region (3′UTR) of GLW7 includes a recognition site for miR156. As expected, overexpression of *miR156* reduces *OsSPL13* expression and leads to a small grain size phenotype (Si et al., 2016). OsSPL13 also binds the *Small and Round Seed 5* (*SRS5*) gene promoter and promotes SRS5 expression to control glume cell size. *SRS5* encodes an α subunit of tubulin protein that forms cellular microtubes. *srs5* mutants display reduced glume cell length with small and round seeds. Overexpression of SRS5 significantly increases grain length. The miR156-OsSPL13/GLW7-SRS5 grain regulation pathway has been well established (Segami et al., 2012, 2017). A recent study that suggested OsSPL18 is cleaved by OsmiR156k. The study also revealed that *OsSPL18* knockouts exhibit reduced grain width and thickness, reduced panicle length and reduced grain number; however, they also exhibit increased tiller number. OsSPL18 binds the *OsDEP1* promoter, suggesting that a novel OsmiR156k-OsSPL18-DEP1 pathway exists in rice (Yuan et al., 2019).

#### Other miRNAs and Their Targets Affect Grain Traits

*Laccase-like* (*OsLAC*) is a negatively regulated target of OsmiR397. Both genes are predominantly expressed in young panicles and grains. OsmiR397 overexpression lines show significant increases in grain length, grain width, thousand grain weight and grain numbers per spikelet. RNA-seq data suggests overexpression of *OsmiR397* and *OsLAC* alter expression of BR signaling-related genes. Overexpression of *OsLAC* causes lethal defects and semi-sterile phenotypes. Therefore, OsmiR397 downregulates OsLAC expression to enhance BR signaling, subsequently increasing yield production (Zhang et al., 2013). In addition to OsmiR397, the isoform OsmiR397b also targets OsLAC. OsmiR397b is regulated by OsAGO17 to control grain weight and grain size, thus establishing an AGO17-OsmiR397b-OsLAC regulation pathway (Zhong et al., 2020).

OsmiR396 regulates multiple production traits including panicle numbers and grain size by regulating the transcription factor Growth Regulating Factor (GRF). Multiple independent studies have revealed miR396-GRF-GIF (GRF-interacting factors) regulation mechanisms (Tsukaya, 2015). *OsGRF4* encodes a transcription factor containing a Gln-Leu-Gln (QLQ) and a Trp-Arg-Cys (WRC) domain that positively regulates cell division and cell elongation (Hu et al., 2015). OsGRF4 mRNA contains an OsmiR396 binding site. A mutation changing TC to AA in exon three abolishes binding and cleavage by OsmiR396, leading to increased cell division and cell elongation as well as significantly increased panicle length, grain length and thousand grain weight. Additional evidence suggests that OsGRF4 and OsGIF1/2/3 interact in young panicles. Overexpression of OsGIF1 mirrors overexpression of OsGRF4 in terms of grain size phenotypes, suggesting OsGRF4 coordinates with OsGIF1/2/3 to modulate grain size and yield production (Duan et al., 2015; Li et al., 2016). OsGRF4 induces expression of *CKX5* and *CKX1* to regulate CK concentration *in vivo* (Sun et al., 2016). In addition to the CK pathway, OsGRF4 also interacts with GSK2, a key negative regulator of BR signaling, to regulate grain size (Che et al., 2015). The OsmiR396 family members *OsmiR396e* and *OsmiR396f* also reduce GA precursor and *CYP96B4* expression independently to affect grain size and plant architecture (Miao et al., 2020).

Short Tandem Target Mimic (STTM) has been used to block the interactions between *OsmiR159* and its target genes. Applying this method increased the expression of the *OsmiR159* target genes MYB, OsGAMYB, and OsGAMYBL1, among others. Furthermore, changes in the expression of genes related to the phytohormone signaling caused multiple phenotypes including changes in plant height, leaf length and grain size (Zhao et al., 2017).

*OsmiR408* is the most conserved microRNA in plants. Overexpression of *OsmiR408* significantly increases the concentration of copper ions in chloroplasts, elevates plastocyanin, and induces photosynthetic genes. This suggests that *OsmiR408* is a positive regulator of photosynthesis and shows great potential for crop applications seeking to improve production yield (Pan et al., 2018).

**FIGURE 3:**
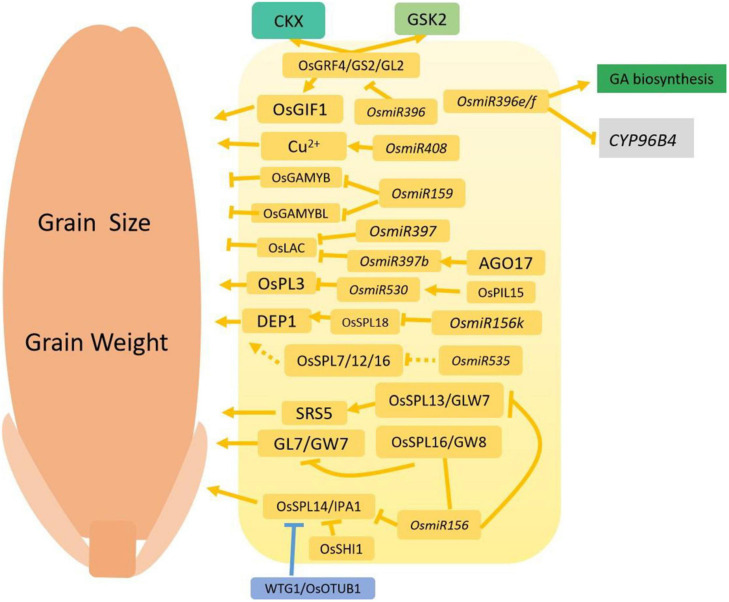
Regulation networks involving microRNAs functioning in grain size and grain weight. Normal arrows indicate the enhanced or positive regulation, and the block arrows indicate down-regulation, or negative regulation. The solid lines represent interactions supported by scientific evidence, and the dotted lines represent hypothetical associations. Yellow indicates the proteins that are involved in microRNA signaling pathways. Blue indicates the involvement of ubiquitin-mediated proteasome degradation, and green colored boxes represent the phytohormone pathway.

*OsmiR535* is highly expressed in young panicles and represses the expression of *OsSPL7/12/16* as well as downstream genes such as *OsPIN1B*, *OsDEP1*, *OsLOG*, and *OsSLR1*. This suggests that *OsmiR535* modulates plant height, panicle architecture and grain shape possibly by regulating rice OsSPL genes (Sun et al., 2019). OsmiR530 is also regulated by OsPIL15 and targets the PLUS3 domain-containing protein (OsPL3) to regulate grain size (Sun et al., 2015, 2020). Figure 3 shows the complex regulatory networks involving microRNA-related processes (Figure 3).

### Other Processes Involved in Grain Weight

*GS5* is a major QTL for grain width and grain weight and is located on the short arm of chromosome 5. GS5 encodes a putative serine carboxypeptidase and positively regulates grain size. Transformation assays and GWAS analyses suggest that variations in the GS5 promoter diversity leads to changes in GS5 expression and further affects expression of genes involved in the G1 to S cell cycle transition (Li et al., 2011).

*LG3* encodes a nuclear AP2 transcription factor and positively regulates grain size. *OsLG3-RNAi* lines have reduced grain length, while OsLG3 overexpression lines have increased grain length. Phylogenetic analysis of *OsLG3* suggests that the *OsLG3* gene arose independently in *O. sativa japonica* and *O. sativa indica* (Yu et al., 2017).

## Integrating Structural Information to Accelerate Molecular Design Breeding in Crops

The rapid growth of the field of structural biology in the past decades has greatly benefited particularly studies involving high throughput drug screening and microorganisms engineering, which have entered a new era. However, studies combing plant protein structural and functional information has remained largely unexplored. We performed a database search for protein structural information related to grain weight in rice (Supplementary Table 1). Among the total of 127 proteins discussed in this current review, only 10 of them could be associated with structural information (including four identical proteins and six homologous proteins). Structures of 73 of the homologous proteins in plants were solved, and the sequence-based search for 31 proteins did not produced any results in the Worldwide Protein Database Bank (wwPDB) database. The huge gap in this field provides us a promising study direction for combining structural information with functional studies. Some recent examples provide a glimpse of an exciting and bright future for these interdisciplinary studies. In 2014, a group from Japan solved the structure of OsRac1 and elucidated the binding sites for NADPH oxidase OsRbohB and the mechanism of ROS production in rice cells using X-ray crystallization and Nuclear Magnetic Resonance (NMR) methods (Kosami et al., 2014). Another example is the crystal structure of gibberellin 2-oxidase 3 (OsGA2ox3), and auxin dioxygenase (DAO) and their allosteric mechanism toward GA4 and IAA. Both GA2ox3 and DAO form multimers and interact with their substrates. Evidence shows that multimerized enzymes have higher specific activities than monomer forms in maintaining stability of GA and IAA levels (Takehara et al., 2020). These examples reveal the possibility of improving these traits by modifying the binding sites using gene editing engineering.

These structural studies have also emerged in other plants. Bastet et al. (2019) investigated eukaryote initiation factor 4E (eIF4E), which confers pathogen resistance of potyviruses in many crops. Several alleles of *eIF4E1* in *Pisum sativum* were introduced into Arabidopsis Col-0 and *4e1*^KO^ lines. They assessed the role of these proteins on eIF4E1 structure and transformed them into Arabidopsis to determine the function of these proteins and the potential pathogen resistance toward multiple potyviruses. The authors even demonstrated how a single mutation, N176K, to transgene-free plants using base editing technology increased Arabidopsis resistance to *Clover yellow vein virus* (ClYVV) (Bastet et al., 2019). This paper showed how the combination of structural studies and accurate gene editing technology benefits plant pathogen resistance.

**FIGURE 4:**
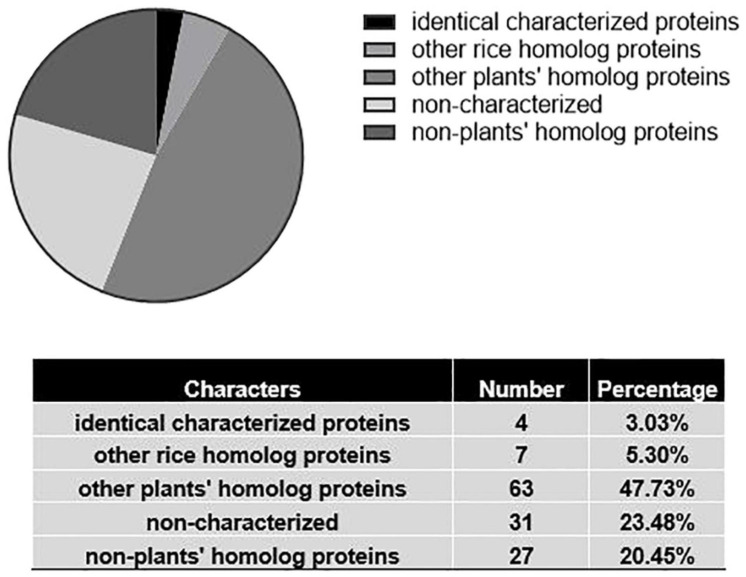
Structural information on grain weight proteins in rice. Results of sequence-based search of the wwPDB database (April 2021). Only about 3% of proteins had structure information of identical proteins. 23.5% of protein structures did not have any equivalents among known proteins of any species that were already characterized.

Another exciting example is the discovery of ligand recognition motifs of LysM domains in nodulation plants (Bozsoki et al., 2020). The authors solved the structures of chitin-mediated receptor CERK6 and nodulation-receptor NFR1 in *Lotus japonicus*. The key LysM1 domain of the two proteins contributed not only to immune reaction but also nodulation. The LYK3 in *Medicago truncatula* extracellular domain’s crystal structure illustrated the unique function for nod factor and ligand recognition. The paper provides a breakthrough and novel insights on enzymatic engineering in non-nodulation plants acquiring the ability of nodulation. According to the information we gathered, among 132 proteins, which is less than 77% of the proteins structural information, has been reported; 3.03% of rice homolog proteins were studied, and 47.7% homolog proteins in other plants (mainly *Arabidopsis thaliana*). Surprisingly, the structure of more than 23.48% of proteins has not been studied (Supplementary Table 1 and Figure 4). Thus, there are still large gaps remaining for deciphering structural information in rice as well as other crops.

In summary, the two examples illustrate how using structural information and gene editing tools can guide targeted molecular breeding in crops, which would largely improve man-made QTL selection with certain traits.

## Conclusion and Perspective

Grain development involves numerous molecular and biological processes related to chromatin modification, transcriptional regulation, translational modification and protein interaction, as well as metabolomic processes related to photosynthesis, accumulation, transportation, distribution and storage, and cellular processes related to cell division, cell differentiation and cell nucleus alteration. Plant activity, which includes all synergistic and antagonistic cell modulation activities from phytohormone biosynthesis and signaling transduction, affect grain shape. Different biotic and abiotic stresses affect *in vivo* phytohormone concentrations and subsequently influence the distribution and accumulation of photosynthetic products in addition to altering cell division, cell expansion and cell differentiation. The trade-off between grain number and size has been well elucidated (Guo et al., 2018), marking an important concept that the grain weight has a weak or even a negative correlation with grain yield. This association is obviously appeared in *japonica* varieties. Interestingly, a study performed by Li R. et al. (2019) concluded that associations between agronomic traits and yield were ecotype-dependent. They collected 7,686 public inbred rice and hybrid rice traits released in 1978–2017 and monitored their agronomic traits associated with grain yield. They found that greater filled grain numbers per panicle, 1000-grain-weight, plant height, panicle length, grains per panicle, and seed setting rate, longer growth period, lower panicle number per unit area, and lower seed length/width ratio accounted for higher yield in *indica* inbred and *indica* hybrid, while only high panicle number per unit area and longer growth period led to higher grain yield in *japonica* inbred and *japonica* hybrid varieties. This conclusion provides breeding hypothetical guidance on various rice ecotypes (Li R. et al., 2019). Another similar study in China focusing on grain quality in 635 rice varieties from 2000 to 2014 revealed different correlations in chalky rice rates and chalkiness degree among *indica* and *japonica* varieties (Feng et al., 2017). These papers suggested that breeding strategies should be altered based on ecotypes and consumption area. Noticeably, the large-grain rice cultivar Akita 63 yield characteristics was analyzed in comparison with its maternal and paternal cultivars (Makino et al., 2020). The trade-off between single grain weight and grain number was found for maternal variety Oochikara but vanished in Akita 63. The successful breeding application of *GS3* and *qSW5* broke the trade-off and had a great impact on yield with improved N-use efficiency, emphasizing the increased potentials of sink and source capacity in grain yield improvement. The same conclusion that sink-source relationships fundamentally contribute to seed size was also mentioned in a recently published review (Li et al., 2021). In summary, grain development is a complicated process involving multiple regulatory levels.

As the most consumed crops, molecular studies of grain weight have been intensely pursued over the past decades. Even through many conclusions have been made, the multiple factors that determine grain weight have yet to be fully elucidated. Recently, novel regulatory pathways related to plant development and environmental adaptation have been described in Arabidopsis and rice. These include the mRNA modification pathway (m6A, m5C modification etc.) (Duan H. C. et al., 2017; Ruzicka et al., 2017; Anderson et al., 2018; Zhang F. et al., 2019; Tang et al., 2020; Wu et al., 2020), tRNA modification pathway (methylation, phosphorylation etc.) (Bouvier et al., 2006; Chen et al., 2019a; Guo et al., 2019; Jin et al., 2019), and post-translational regulation pathways (Zhao et al., 2020; Qi et al., 2021). The reports on certain genes hint at the possibility that these pathways are involved in grain weight. Illustrating the functions and applications of these genes in crops will shed new light on the agronomic development. Meanwhile, successful breeding efforts in rice have the potential to also benefit other monocot crops, such as wheat and maize. A famous example is *Maize Floury 3* (*FL3*), which encodes a plant AT-rich sequence and zinc binding (PLATZ) protein. FL3 interacts with RNA polymerase III subunit C53 (RPC53) and transcription factor class C1 (TFC1) of the RNAPIII complex to modulate the RNAPIII transcription machinery (Li Q. et al., 2017). Interestingly, a similar regulation pattern was identified in rice as grain length QTL GL6, is a homolog protein of FL3 in maize (Wang A. et al., 2019). Researchers in rice or other plant species should focus on exploring the novel and mutual mechanisms, and this direction could further guide agronomy to broader horizons.

Since the original paper describing the use of CRISPR gene editing in rice was published (Shan et al., 2013), numerous papers have focused on how to use gene editing as a precise tool to accelerate crop breeding (Chen et al., 2019b; Moradpour and Abdulah, 2020). Some reports have described CRISPR methods for introducing single or multiple base pair changes in the promoter of relevant genes in an effort to confer new traits (Zhang H. et al., 2018; Zsogon et al., 2018). Moreover, promoter gene editing using CRISPR-Cas9 systems has been reported to create QTLs in plants, demonstrating the perfect combination of this technology with agriculture applications (Wang et al., 2021; Xu et al., 2021). This suggests that CRISPR related technologies may be feasible tools for future molecular breeding. As many important QTLs and genes have been identified and their mechanisms revealed, it may be possible to apply CRISPR technology in these regions to create novel rice QTLs. This research will also be important for other global crops. As more molecular mechanisms of grain traits are discovered and molecular breeding techniques are applied, “design breeding” will position itself within modern agriculture and provide great benefits to all humankind.

In this review, we have summarized most quantitative and qualitative trait loci associating with rice grain weight. Within the past decades, many single genes and QTLs were cloned and characterized, building up fundamental regulation networks controlling these traits. However, simply pyramiding positive alleles related to grain size determination is not the best solution to improve grain yield and quality. The trade-off beneath these genes and pathway existed and need better coordinating in future molecular breeding. Theoretically, grain weight merely determines an isolated plant yield production, and it is independent and not predictive to a dense crop standing. However, grain size, as well as seed quality, is typically stable despite the fact that environmental and nutrient conditions are also key determinants. A potential direction is to figure out all the possible expression pattern of certain genes in grain morphogenesis. Using genome data as well as phenotypical data to investigate the best harmonized expression patterns of regulation networks group would deepen our understanding of grain size and quality determination factors. The optimized grain development process should meet the best carbon and nitrogen nutrient flow from sink to source. Recently a new pan-genome analysis of 33 genetically diverse rice accessions revealed multiple structural variations and gene copy number variations information (Qin et al., 2021). To utilize this variation information as well as phenotypical messages, researchers should accelerate molecular breeding work on these important traits. Hybrid breeding and gene editing on promoter regions to change the expression pattern of certain genes is one option toward precision agriculture.

## Author Contributions

All authors listed have made a substantial, direct and intellectual contribution to the work, and approved it for publication.

## Conflict of Interest

The authors declare that the research was conducted in the absence of any commercial or financial relationships that could be construed as a potential conflict of interest.

## Supplementary Material

Supplementary Table 1 The structural information of genes related in the paper. Data was collected from Phytozome database (https://phytozome.jgi.doe.gov/pz/portal.html) and Protein Data Bank in Europe (https://www.ebi.ac.uk/pdbe/).
